# Global Renewable Energy-Based Electricity Generation and Smart Grid System for Energy Security

**DOI:** 10.1155/2014/197136

**Published:** 2014-08-27

**Authors:** M. A. Islam, M. Hasanuzzaman, N. A. Rahim, A. Nahar, M. Hosenuzzaman

**Affiliations:** UM Power Energy Dedicated Advanced Centre (UMPEDAC), Level 4, Wisma R&D University of Malaya, Jalan Pantai Baharu, 59990 Kuala Lumpur, Malaysia

## Abstract

Energy is an indispensable factor for the economic growth and development of a country. Energy consumption is rapidly increasing worldwide. To fulfill this energy demand, alternative energy sources and efficient utilization are being explored. Various sources of renewable energy and their efficient utilization are comprehensively reviewed and presented in this paper. Also the trend in research and development for the technological advancement of energy utilization and smart grid system for future energy security is presented. Results show that renewable energy resources are becoming more prevalent as more electricity generation becomes necessary and could provide half of the total energy demands by 2050. To satisfy the future energy demand, the smart grid system can be used as an efficient system for energy security. The smart grid also delivers significant environmental benefits by conservation and renewable generation integration.

## 1. Introduction

**Figure 1:**
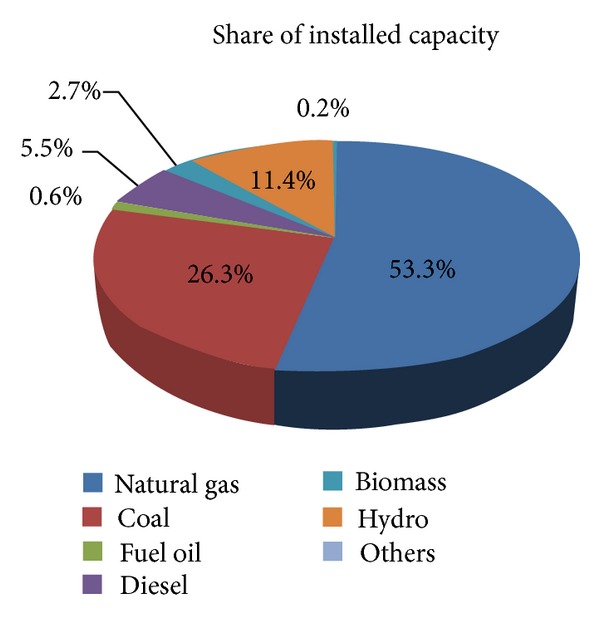
Share of installed capacity as of December 31, 2012, in Malaysia [88].

Economic growth, automation, and modernization mainly depend on the security of energy supply. Global energy demand is rapidly growing, and, presently, the worldwide concern is on how to satisfy the future energy demand. Long-term projections indicate that the energy demand will rapidly increase worldwide. To supply this energy demand, fossil fuels have been used as primary energy sources. Fossil fuels emit greenhouse gases that highly affect the environment and the future generation [1–6]. The emissions largely depend on the emission factor of primary energy sources (i.e., input fuel of the plant). Among all energy sources, the emission factor of fossil fuels (i.e., coal, natural gas, and oil) is very high, as shown in Table 1. Fossil fuels are widely used as the main fuel in power generation. In Malaysia, fossil fuels (i.e., natural gas [53.3%] and coal [26.3%]) serve as major power generation sources, as shown in Figure 1. Large-scale use of fossil fuels, however, greatly affects the environment. Based on the global CO_2_ distribution in 2013, the emission breakdown is as follows: coal (43%), oil (33%), gas (18%), cement (5.3%), and gas flare (0.6%) [7].

Meanwhile, renewable energy sources (solar, wind, hydro, geothermal, biomass, etc.) are emission-free energy sources in the world. Renewable energy technologies are an ideal solution because they can contribute significantly to worldwide power production with less emission of greenhouse gases [8–11]. The “sustainable future” scenario of the International Energy Agency (IEA) shows 57% of world electricity being provided by renewable energy sources by 2050 [12]. Long-term forecast and planning is required to achieve this ultimate target [9]. Renewable energy-based power generation and supply to the national grid for a specific zone are necessary. The conventional grid aggregates the multiple networks, and the regulation system consists of various levels of communication and coordination, in which most of the systems are manually controlled [13]. A smart grid is a new concept that leads to the modernization of the transmission and distribution grid. The smart grid system is the digital upgrade of transmission and new markets for the alternative energy generation of renewable energy sources. Presently, smart grid is an often-cited term in the energy generation and distribution industry [14].

Smart grid connected with distributed power generation is a new platform that significantly generates reliable security of supply (SOS) and quality of electric energy. This concept is practical and reliable as numerous types of energy sources become available, such as solar, wind, biomass, and hydropower. Renewable and nonconventional energy sources are allowed to integrate with the distributed power generation link that has a smart grid. This study therefore highlights the role of renewable energy sources in generating electricity and the integration with the smart grid system for energy security.

## 2. World Energy Consumption Scenario 

Global population growth and improvement of living standards cause high energy demand [15]. This global energy demand is increasing faster than the population growth rate [16]. Approximately 80% of the total primary energy is being supplied by fossil fuels [17]. World energy consumption projection from 2002 to 2030 shows the increase of energy demand by almost 60% (1.6% per year). The energy demand will be approximately 16.5 billion tons of oil equivalents (toe) by 2030 compared with 10.3 billion in 2002 [18, 19]. Tables 2 and 3 present the projection of the world primary energy demand from 2002 to 2030.

The global primary energy consumption projection shows that fossil fuels will solely contribute the largest amount of energy, not taking into consideration if its share will slightly decrease from 36% in 2002 to 35% in 2030 [20]. The demand for gas in the power sector will tremendously increase to a maximum of 60% by 2030, and the market share will increase up to 47% by 2030 compared with 36% in 2002. In most parts of the world, natural gas is expected to remain as a main competitive fuel in the new power station because of its high efficiency [21–23]. The share of coal in fulfilling the total primary energy demand will decrease from 30% in 2012 to 27% by 2035 [24]. The nuclear energy will decrease to 5% by 2030 from 7% in 2002. The intensities in different regions will continue to vary because of levels of variation in economic growth, energy use of different users, energy prices, geography, economic arrangement, culture, lifestyle, and climate [19, 21–23].

## 3. Sources of Renewable Energy for a Green and Clean World

**Figure 2:**
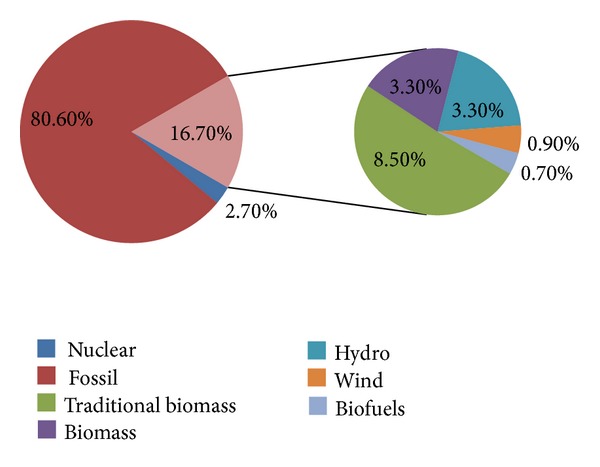
Global energy consumption and share of renewable energy, 2010 [89].

**Figure 3:**
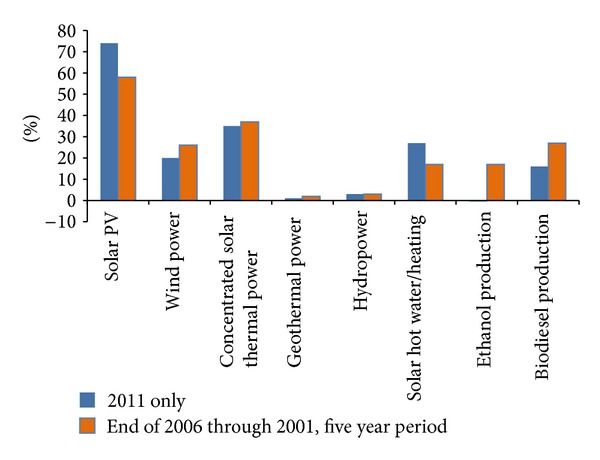
Renewable energy capacity growth rate, 2006–2011 [89].

**Figure 4:**
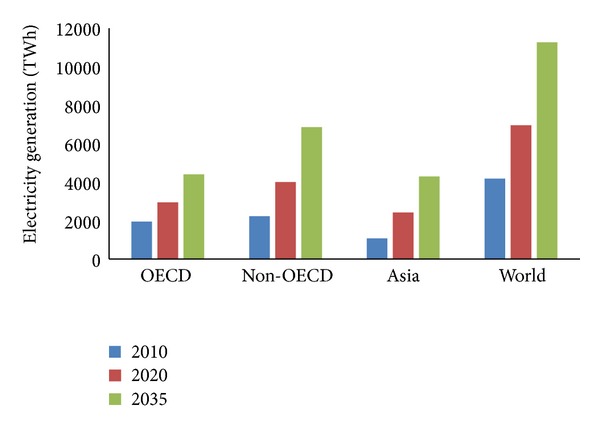
Renewable-based electricity generation by region [36].

Adequate energy sources and supply are essential for the economic and social growth of any nation. The development of the energy supply system is the main objective of the United Nations Millennium Declaration. In developed countries, innovative technologies and modern concepts have to be upgraded to enhance energy efficiency, which is considered as the performance indicator of the millennium declaration. From an international perspective, renewable energies provide several benefits to the conventional energy system. Renewable energy reduces CO_2_ emissions, which is the main aim of climate protection. Renewable energy reduces dependency on fossil fuels and energy import from other countries and improves economic growth [18, 25–31]. Presently, enormous challenges are being encountered regarding the necessities of life. Moreover, service requirements continuously increase with the increase of power consumption. According to IEA [32] in Paris, by 2030, approximately 60% of energy utilization will increase compared with the utilization level in 2001. Fulfilling the energy demand by using fossil fuels as primary resource would be more difficult. Power generation from fossil fuels can potentially harm the environment and can cause global warming. Thus, the next-generation energy system must be sustainable and carbon-free. Policy makers should promote renewable energy (i.e., solar, wind, biomass, hydropower, and geothermal) as primary sources of energy. The potentiality of the current renewable energy technology is extensive and positive. Review of the literature shows that half of the total energy demands could be satisfied by renewable sources by 2050 [25, 33, 34]. The share of renewable energy sources is predicted to contribute approximately 30% to 80% in 2100 [35]. The total energy share of various fields of energy sources and particularly the contribution of renewable energy (16.70%) sources of the total world energy consumption is shown in Figure 2. The annual average growth rate of the capacity of the world renewable energy between 2006 and 2011 is shown in Figure 3.  Table 4 shows the summary of world renewable energy use by type and scenario. The renewable energy-based electricity generation is increasing; that is, the share of total electricity generation was 20% in 2010 and will be 31% by 2035.  Figure 4 shows that the rate of renewable energy-based generation in Organization for Economic Cooperation and Development (OECD) is higher (approximately 18% in 2010 and 33% by 2035) than that in other regions [36].

The renewable energy-based electricity generation has been increasing where the share of total electricity generation was 20% in 2010 and will be 31% by 2035.  Figure 4 shows that the rate of renewable energy-based generation in OECD is higher (about 18% in 2010 and 33% by 2035) than other regions [36].

## 4. Smart Grid and Energy Security

To improve the sturdiness and reliability of the grid, the security systems should be improved in both the physical and cyber perspectives. Ultimately, this action will reduce the probability and consequences of man-made occurrences. Energy security is a concept that ensures the reliability of energy sources, maintains a sufficient energy supply at an affordable price, and prevents the harmful effects to the environment. Energy security is a multidimensional issue that addresses risk management, diversity of energy, and decision making for implementing the policy [37]. The integration of renewable energy using smart grid technologies can improve energy security and safety of the electric system.

### 4.1. Smart Grid

**Figure 5:**
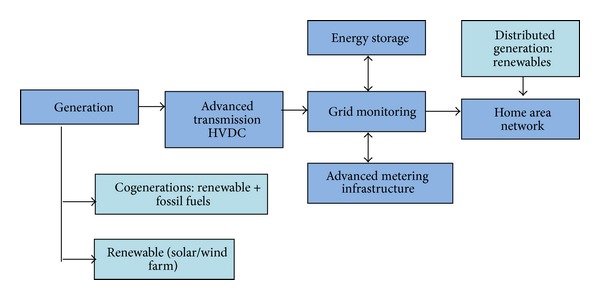
Block diagram of the smart grid concept [86].

A smart grid is the solution to the modernization of the electrical energy system and infrastructure to present a more intelligent and reliable electricity grid. Smart grids provide many benefits over conventional grid. Smart grids improve both the physical and economic operations of the grid system, increasing reliability and sustainability [38]. A more conceptual definition of smart grid is presented by Rahman [39]. According to the modern technology-based grid initiative of the United States Department of Energy, an intelligent self-response is based on the demand or a smart grid integrating and combining with advanced sensing, monitoring technologies control methods, and two-way communications into the current electricity grid.  Figure 5 shows the block diagram of the smart grid concept. Energy security and optimization of the demand can be minimized by implementing the smart grid system.

**Figure 6:**
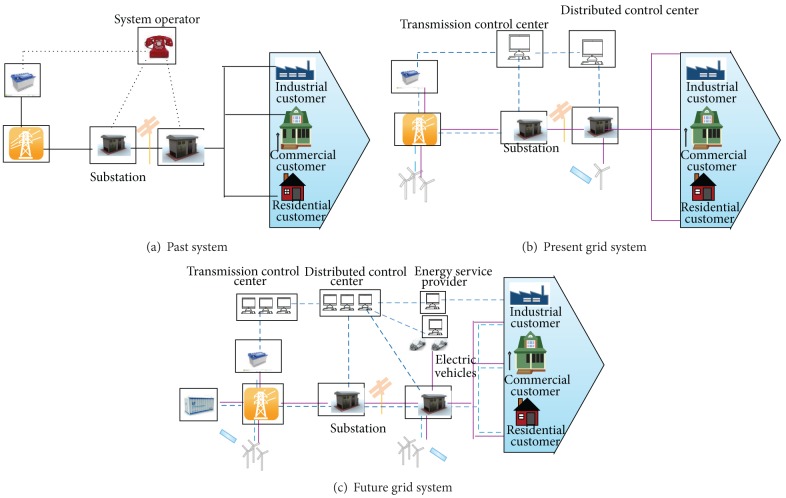
The comparison between conventional grid and smart grid [90].

The smart grid system is designed to handle uncertain incidents. The three security objectives of smart grid are to ensure (1) the availability of the power supply based on customer requirement, (2) the two-way communication system, and (3) the data security of the customer [40]. The smart grid mainly aims to enhance overall management, which refers to obtaining better control of the transmission system that will improve system reliability. This technique has numerous advantages with regard to frugality, despite the low energy efficiencies (system losses occurring along the distribution line). Smart grid technologies are capable of supporting the system operator in controlling and managing the energy streams on the grid with more accuracy by applying the flexible AC transmission systems. First, using a modern sensor that is called a phasor measurement unit that determines the real-time response of service providers, the efficiency of the total electricity system is improved [41]. Second, the automation of the smart grid will be more self-responsive, and better control of the substation on the distributed network is ensured. The distribution system automation of the smart grid allows utility firms to upsurge the strong communication of the distribution network and prevents the interruption of supply to the end user at unanticipated incidents such as an environmental hazard that destroys power poles or causes infrastructure damage to the substation. The end-user load is also controlled by implementing the distribution channel automation. The integration of modern communication technology with various grid segments provides better and reliable service to the end user, which is the basic role of the smart grid.  Figure 6 shows the comparison between conventional and smart grids.

**Figure 7:**
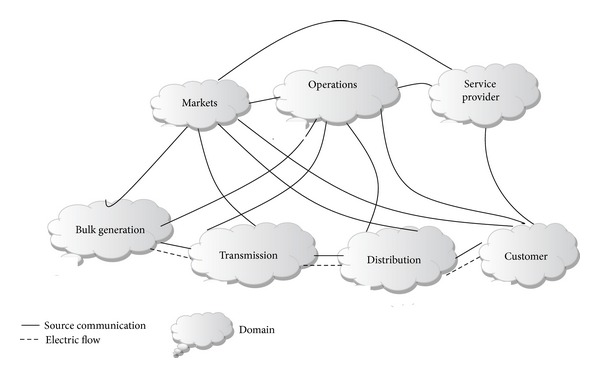
NIST smart grid domains through secure communication flows and electrical flows [91].

Figure 7 shows the smart grid domains through secure communication and electrical flows. This control system logically increases the confidential issues of individual end-user level information [42]. The peak load time of end-user appliances is automatically recognized by a smart metering system, without employing a service person to collect the data from the electric meter.

#### 4.1.1. Smart Grid Technology and Applications

Modern engineering tools and techniques are required to develop the smart grid. The integration of information technology, strong monitoring system, and practical strategic plan is necessary to completely understand the smart grid application. The demand for electricity being satisfied by the centralized and distributed generation (DG) system through the use of a smart grid technology is a very modern and reliable concept. The system operation and control system of the smart grid are monitored by modern information and communication technologies that enable the operator to practice control over the demand and efficiently provide reliable and high-quality service. The smart grid provides the most effective electrical distribution network through the two-way communication system based on the responses of the customer. Power industries worldwide are unpredictably facing huge challenges. Existing grids are also challenged to perform safely and provide reliable supply. In addition, social and political gain is important and depends on the electricity generation and utilization and its environmental impacts [43]. Developing countries are formulating their policy based on the requirement of an enhanced smart grid. A huge amount of federal funding has been allocated to promote and assist the smart grid policy in different states. Better management for the smart grid in the electricity industry is time-consuming. The smart grid is integrated into the infrastructure that supplies electricity, which is coupled with modern telecommunication, IT, and sensing technology. The great potentiality of the smart grid is defined by its capability to process and to analyze a huge amount of data and to implement critical demand management. The smart grid provides flexible opportunity to the system operator and the end users with its use of artificial intelligence and integration with the computer system. The application of the smart grid in developed countries is happening much quicker than its display of numerous benefits [44]. The end user is the dynamic player in the electricity industry. Benefits and savings can be attained by optimizing peaks in demand and by increasing the energy performance. The successful application of smart grid is the key factor in reaching its ultimate aims to reduce greenhouse gas discharges and to utilize energy efficiently [45].  Table 5 shows the smart grid technologies, applications, and purposes. Demand management would be quicker if the network would connect with the innovation, new energy products, and services. As a result, the information channel, statistics storing and management, and rules of governing access by various customers are developed. The eight priority areas in building a smart grid (as identified by the National Institute of Standards and Technology (NIST)) are as follows [46]: (a) demand response and consumer energy efficiency, (b) wide-area situational awareness, (c) energy storage, (d) electric transportation, (e) advanced metering infrastructure, (f) distribution grid management, (g) cyber security, and (h) network communications.

#### 4.1.2. Reliability of the Smart Grid

Reliability issues in modern power grids are becoming more challenging. The challenges include aggravated grid congestion, larger transfers over longer distances, increasing volatility, and reduced reliability margins [47]. The integrated network of islanding connected with DG could increase reliability and enhance the good quality service of the local electricity supply. The electrical charge based on real-time sensing is practical for the consumer; this charge is possible by implementing advance metering [48]. The smart grid possesses particular characteristics or delivers the following: it self-heals from power disturbance events, permits end-user participation in demand management, robustly manages physical damage and cyber-attacks, provides the power quality required in the 21st century, is flexible in accepting all generation and storage options, permits the introduction of new products, services, and markets, and develops the operating efficiency and optimization of assets [41]. Smart meters now account for 85% of all such devices in Italy and 25% in France. Many governments have aimed at nationwide placements of smart grids by 2020 [49]. Developed countries also envision the amplification and application of energy or climate protection policies and solve problems relevant to them [50]. Each country has its specific viewpoint in gaining the benefits of the market segment from smart grids.

### 4.2. Energy Security

The focus on energy security and reliability is based on the concept that a continuous supply of energy is critical for an effective economy of any country. The meaning of security of energy or SOS varies among different people at different places [51]. The SOS of energy is mainly associated with the security of access to oil or gas supplies and is connected with the future scenario of maintaining the fossil fuel reserve of a particular nation. Every day the definition and concept of energy security change and are modified by the changes in technology. Presently, energy security is defined by four main and basic elements [52–55]. Security of energy involves the readiness or real existence of fossil fuels, gap, and discrepancy between energy consumption and generation, cost involved with the SOS, and environmental sustainability (e.g., related to the obtainability of solar, wind, and bioenergy). The Asia Pacific Energy Research Center [54] has identified and classified the elements that are related to SOS as follows:

- accessibility or geopolitical elements;
- acceptability or environmental and societal elements;
- availability or elements relating to geological existence;
- affordability or economical elements.

### 4.3. Indicators of Energy Security

Energy security is an important factor for economic development and consists of several relevant factors that are aggregated using variables. Energy security indicators explain the contribution of SOS. Most of the indicators are subjected to a particular context. An individual indicator has particular significance. Thus, understanding the suitability and application of dissimilar SOS indicators is important. SOS indicators are used in analyzing the scenario of energy SOS under different perspectives [56].

#### 4.3.1. Energy Resources and Import Dependency

Energy reserve, where energy resources are considered as direct indicators, is vital for SOS [56]. Reduction and adjustment of energy demand can contribute to energy security. Generally, residential and business areas with tolerable demands are easier to supply and are less exposed to energy price increases. Thus, the tolerable energy demand of any place can cause few opportunities for energy to be imported from other countries. The demand for energy involves the following: energy demand per home or unit of economic activity, energy costs as a proportion of total expenditure that indicates the severity of exposure to price increases, and capacity for demand-side response [57]. The existence of hydrocarbon resources is very uncertain and unpredictable, and modern technology is necessary to explore these resources. The United States Geological Survey [58] is one of the famous organizations that provide the most authentic information relevant to geographical resource estimation. The data provided by this organization are considered as the most recognized, reliable, and autonomous [59]. The most frequently used SOS indicator is import dependence. This indicator refers to the importation of oil as being dependent on other regions and is often relative to oil consumption [60]. This indicator is commonly used in measuring energy security; that is, high import dependence means low energy security [56].

#### 4.3.2. Indices of Energy Diversity

Diversity of energy lessens the probability of disruption of energy supply. However, how an energy system may be diversified and how it can be measured remain unclear. Diversity index is a combination of several concepts, such as energy resources used by an economic sector or country, countries and/or companies supplying those resources, and technologies and infrastructure used to convert, transport, and deliver energy to consumers [57]. Diversity of energy sources in various geographical locations is considered as hindrance to supply risks [61, 62]. A statistical quantity of the multiplicity of energy sources is an indicator of SOS. The indicator of diversity consists of three basic factors [63]: variety (number of sets), balance (range across types), and disparity (individuality that makes groups different from one another). However, no index can measure appropriate diversity. Diversity index means measuring the multiplicity or diversity in energy supply, not taking into consideration if the classification is still influenced by the individual option. This formal indicator is not considered as the thread of trouble created by various fuels [56].

#### 4.3.3. Political Stability

The political situation of the country that supplies energy is an important factor in the security of energy supply. Energy supply actually depends on the negotiation between the governing body of the country and other parties. The political risk indicators are mentioned by the World Bank, such as political stability, absence of violence, and regulatory quality [18, 53, 56, 64]. The political indicator is a geopolitical market concentration risk. Political factors associated with the exporting countries are measured by the political indicator. Low value of geopolitical market concentration indicates high political risk [65].

#### 4.3.4. Price of Energy

The demand and supply balancing mechanism is a common function and is influenced by market prices. Prices are affected by the supply in relation to demand; thus they are recognized as a measure of economic impacts. The oil price is an important indicator of SOS because it is affected by many factors (i.e., speculation, strategic communication, and short-term shortages) in the market [56]. The lack of supply affects the market price of energy, and the responses of customers contribute to the market equilibrium condition. High price of energy leads consumers to reduce their consumption and to search for alternative sources of energy. The price of energy also involves energy security concerns [66].

#### 4.3.5. Market Liquidity

The ratio of the world oil export to the net oil import of the country is called market liquidity. Market liquidity determines the oil availability in the world market against the portion of oil demand in the domestic market that cannot be supplied by the local oil production. Thus, high market liquidity expresses the necessity for extra oil supply from another country [65]. A close relation exists between market liquidity and SOS in balancing the fluctuation of demand and supply of fuels in the market. IEA stated that [62] market liquidity is the exponential function of the ratio of the consumption of a country over the total fuel offered in the market [56, 67].

## 5. Smart Grid and Integration of Renewable Energy Sources

**Figure 8:**
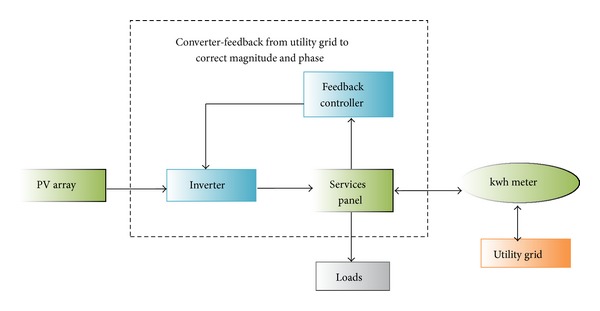
PV-based solar energy integration [92].

**Figure 9:**
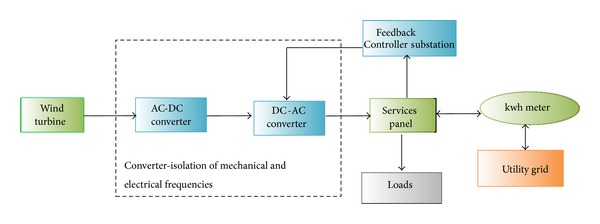
Wind energy integration [92].

**Figure 10:**
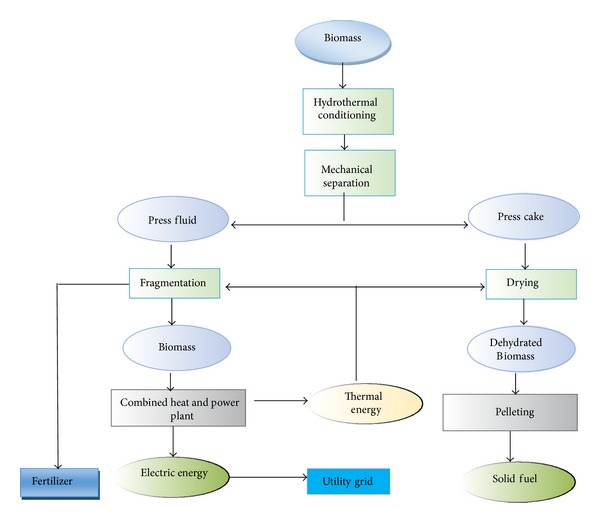
Biomass-based energy [93].

**Figure 11:**
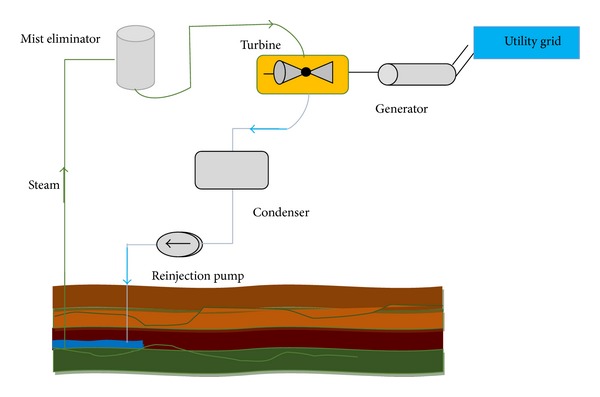
Geothermal-based power generation [94].

Given the rising energy prices and the greenhouse effect, renewable resources are more environmentally convenient and more efficient. Solar technology is the most ideal solution to energy demand management and prevention of greenhouse gas emission and is a milestone to the generation of green and clean energy. The most remarkable technologies involve generation techniques that use wind turbines, solar energy, hydropower, and biomass [68–70]. Figures 8, 9, 10, and 11 illustrate the renewable energy-based integration to the grid.

The extended application of these types of innovation and technologies depends on three crucial factors that are related to the future energy supply and that introduce the multiapplication of grids: DG, distributed energy storage (DES), and demand-side load management (DSLM). In DG, various energy sources are connected to the power grid. These types of sources range from high- to local-level generators such as combined or hybrid power plants [68, 71].

**Figure 12:**
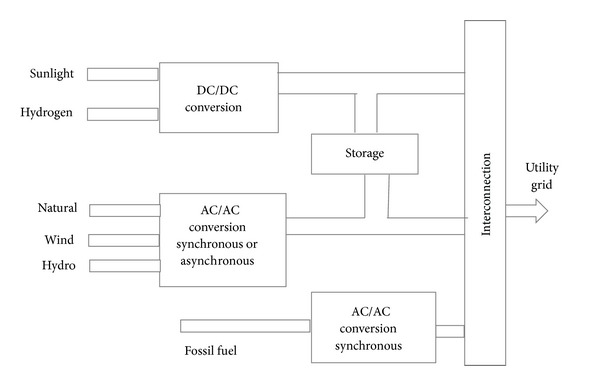
Integration of several sources of energy into the grid.

Local power generation using renewable energy is vital in the implementation of the smart grid [72]. The handling of renewable energy sources requires refined arrangement and operation planning based on the concept of technologies [73]. The large-scale distributed renewable generation system requires a more flexible, reliable, and smarter grid [74]. Energy storage is also the fundamental concept of the smart grid. This system provides support to the development of sustainable energy [75].  Figure 12 shows the overall renewable energy integration and storage system. Given the growing participation of renewable energy to the electricity supply chain, the necessity of energy storage will ultimately increase [68]. The application of a large amount of electricity power storage is challenging and can cause system losses. The application of DES is a possible solution because it slightly reduces the disadvantages of the energy backup requirements of the smart grid. The other benefit of the DES application in a smart grid is the enhancement of DSLM by a small-scale backup policy [76] and the improvement of the generation performance by supporting the peak demand [77, 78]. The local backup of energy is used for the efficient management of addressing energy demand during the peak period. The management of peak demand by efficient and bright management techniques will make the grid reliable and smarter and perform better [75, 76]. Using a hybrid system of power generation will result in a more balanced and controlled management of the grid. As the power generation becomes close to the end user, the hybrid power generation technology provides the service; thus no transmission losses exist [79]. Hybrid generation connected with the smart grid approach has numerous advantages over the conventional system. First, the cost of transmission and distribution is low. Approximately 30% of the cost is involved with electric transmission to other places in a conventional system. The domestic supply line does not have high capital for the initial setup and has less energy losses from long-distance transmission lines. Local connection lines do not have high capital costs and energy losses caused by long-distance distribution lines; energy consumption also decreases because of new innovative devices [80]. Second, DG allows the integration of local renewable energy sources to power plants and is helpful in reducing greenhouse gas emission [81]. The development of hybrid power generation connected with the smart grid reduces emission and satisfies the level of electricity supply against end-user demand [82, 83].  Table 6 shows the potential reductions in electricity and CO_2_ emissions in 2030 attributable to smart grid technologies.

## 6. Conclusion

Ensuring a reliable, efficient, and affordable energy is a great challenge. Generating electricity from renewable energy sources can provide direct and indirect economic benefits in excess of costs as well as environmental benefits through the reduction of CO_2_ emission. Policy makers should promote renewable resources (i.e., solar, wind, biomass, hydropower, and geothermal) for sustainable and carbon-free energy. It is predicted that about 57% of total energy demand could be generated from renewable sources by 2050. The renewable energy source power generation integrated into the smart grid system can be one of the best options for future energy security. The smart grid system addresses the degradation of energy source and modern information technology for communication and improves the efficiency of power distribution. A smart grid can transform the 20th century power grid as a more intelligent, flexible, reliable, self-balancing, and interactive network that enables economic growth, environmental oversight, operational efficiency, energy security, and increased consumer control. Moreover, the smart grid would create new markets as private industries develop energy-efficient and intelligent appliances, new communication capabilities, and smart meters. Smart grid can replace traditional forms of energy with renewable sources of generation. Renewable energy is always required by environmentalists in the hopes of developing a cleaner and more efficient power generation. A smart grid is environmentally beneficial because it utilizes the distribution of renewable sources. Smart grid offers a genuine path toward significant environmental improvement.

## Figures and Tables

**Table 1:** Emission factors of fossil fuels for electricity generation [84].

Fuel	Emission factor (kg/kWh)		
	CO_2_	SO_2_	NO_*x*_
Coal	1.1800	0.019	0.0052
Petroleum	0.8500	0.0164	0.0025
Gas	0.5300	0.0005	0.0009

**Table 2:** World primary energy demand (Mtoe) [18].

Energy sources	2002	2010	2020	2030	2002–2030
Coal	2389	2763	3193	3601	1.50%
Oil	3676	4308	5074	5766	1.60%
Gas	2190	2703	3451	4130	2.30%
Nuclear	692	778	776	764	0.40%
Hydro	224	276	321	365	1.80%
Biomass and waste	1119	1264	1428	1605	1.30%
Others renewable	55	101	162	256	5.70%

	10345	12193	14405	16487	1.70%

**Table 3:** World primary energy demand (%) from 2002 to 2030 [18].

Energy sources	2002	2010	2020	2030
Coal	23%	23%	22%	22%
Oil	36%	35%	35%	35%
Gas	21%	22%	24%	25%
Nuclear	7%	6%	5%	5%
Hydro	2%	2%	2%	2%
Biomass and waste	11%	10%	10%	10%
Others renewable	0.53%	0.83%	1.12%	1.55%

**Table 4:** World renewable energy use by type and scenario [85].

	New policies			Current policies		450 scenario	
	2011	2020	2035	2020	2035	2020	2035
Electricity generation (TWh)	**4 482**	**7 196**	**11 612**	**6 844**	**10 022**	**7 528**	**15 483**
Bioenergy	424	762	1 477	734	1 250	797	2 056
Hydro	3 490	4 555	5 827	4 412	5 478	4 667	6 394
Wind	434	1 326	2 774	1 195	2 251	1 441	4 337
Geothermal	69	128	299	114	217	142	436
Solar PV	61	379	951	352	680	422	1 389
Concentrating solar power	2	43	245	35	122	56 806	56 806
Marine	1	3	39	3	24	3	64
Share of total generation	*20% *	*26% *	*31% *	*24% *	*25% *	*28% *	*48% *

Heat demand∗ (Mtoe)	**434**	**438**	**602**	**432**	**551**	466	704
Industry	209	253	316	255	308	248	328
Buildings∗ and agriculture	135	184	286	177	243	198	376
Share of total demand	*8% *	*10% *	*12% *	*9% *	*11% *	*10% *	*16% *

Biofuels (mboe/d)∗∗	**1.3**	**2.1**	**4.1**	**1.9**	**3.3**	**2.6**	**7.7**
Road transport	1.3	**2.1**	**4.1**	**1.9**	**3.2**	**2.6**	**6.8**
Aviation∗∗∗	—	—	0.1	—	0.1	—	0.9
Share of total transport	2%	*4% *	*6% *	*3% *	*4% *	*5% *	*15% *
Traditional biomass (Mtoe)	744	**730**	**680**	**732**	**689**	**718**	**647**
Share of total bioenergy	*57% *	*49% *	*37% *	*50% *	*40% *	*47% *	*29% *

Share of total renewable energy demand	*43% *	*33% *	*22% *	*34% *	*25% *	*32% *	*17% *

∗Excluding traditional biomass. ∗∗Expressed in energy-equivalent volumes of gasoline and diesel.

∗∗∗International bunkers. Note: Mtoe: million tonnes of oil equivalent; TPED: total primary energy demand; TWh: terawatt-hour; mboe/d: million barrels of oil equivalent per day.

**Table 5:** Smart grid technologies, applications, and purposes [86].

Application	Technology	Purpose
Distributed automation	(i) Alternate energy (ii) Smart sensing (iii) Advanced smart metering	Reduces system losses

Data analysis	Information technology	Collect and analyze from the grid

Demand response	Smart appliances	To achieve lower electricity rates

Carbon management	(i) Alternate energy (ii) Smart sensing (iii) Advanced smart metering	Reduce carbon footprint

Home energy management	(i) Smart sensing (ii) Advanced smart metering (iii) Smart appliances	Track and optimize energy use

**Table 6:** Potential reductions in electricity and CO_2_ emissions in 2030 attributable to smart grid technologies [87].

Mechanism	Reductions in electricity sector energy and CO_2 _emissions∗	
	Direct (%)	Indirect (%)
User information and feedback systems	3	—
Categorization of residential and small/medium commercial buildings	3	—
Energy efficiency measurement and verification programs	1	0.5
Shifting load	<0.1	—
Support electric vehicle and plug-in hybrid electric vehicle	3	—
Advanced voltage control	3	—
Support penetration of renewable energy generation (25% renewable portfolio standard)	<0.1	5

Total reduction	12	6

∗Assuming 100% penetration of the smart grid technologies.
